# Glia Gene Therapy

**DOI:** 10.1002/alz70859_102715

**Published:** 2025-12-25

**Authors:** Or A. Shemesh, Krishnashis Chatterjee

**Affiliations:** ^1^ The University of Pittsburgh, Pittsburgh, PA USA; ^2^ The Hebrew University of Jerusalem, Jerusalem Israel

## Abstract

**Background:**

Glial cells are critical regulators of brain health and disease, playing essential roles in neuroinflammation and homeostasis. Dysregulation of glial activity is associated with neurodegenerative disorders, including Alzheimer’s disease (AD). Genetic factors such as TREM2 have been implicated in modulating glial responses, but effective strategies to selectively modulate glial‐specific pathways remain underdeveloped, representing a significant challenge in addressing glial contributions to disease pathology.

**Method:**

A novel platform was designed to achieve selective modulation of glial activity through the development of advanced delivery systems targeting specific molecular pathways. The platform was evaluated in primary glial cultures and validated in‐vivo in a model of neurodegeneration.

**Result:**

The delivery system achieved precise, sustained engagement of glia‐specific pathways, with minimal off‐target effects in other glial or neuronal populations.

**Conclusion:**

This work establishes a novel approach for selectively targeting glia and modulating pathways implicated in neurodegeneration. By addressing the critical challenge of glial specificity, the platform provides a foundation for future therapeutic development and deeper insights into glial contributions to brain diseases.

